# The Importance of Incorporating At-Home Testing Into SARS-CoV-2 Point Prevalence Estimates: Findings From a US National Cohort, February 2022

**DOI:** 10.2196/38196

**Published:** 2022-12-27

**Authors:** Saba A Qasmieh, McKaylee M Robertson, Madhura S Rane, Yanhan Shen, Rebecca Zimba, Camila A Picchio, Angela M Parcesepe, Mindy Chang, Sarah G Kulkarni, Christian Grov, Denis Nash

**Affiliations:** 1 Institute for Implementation Science in Population Health City University of New York New York, NY United States; 2 Graduate School of Public Health and Health Policy City University of New York New York, NY United States; 3 Barcelona Institute for Global Health (ISGlobal) Hospital Clínic University of Barcelona Barcelona Spain; 4 Department of Maternal and Child Health Gillings School of Public Health University of North Carolina Chapel Hill, NC United States; 5 Carolina Population Center University of North Carolina at Chapel Hill Chapel Hill, NC United States

**Keywords:** COVID-19 prevalence, at-home rapid SARS-CoV-2 tests, population-based surveys, COVID-19, surveillance, public health, rapid test, Omicron variant, point prevalence

## Abstract

**Background:**

Passive, case-based surveillance underestimates the true extent of active infections in the population due to undiagnosed and untested cases, the exclusion of probable cases diagnosed point-of-care rapid antigen tests, and the exclusive use of at-home rapid tests which are not reported as part of case-based surveillance. The extent in which COVID-19 surveillance may be underestimating the burden of infection is likely due to time-varying factors such as decreased test-seeking behaviors and increased access to and availability of at-home testing.

**Objective:**

The objective of this study is to estimate the prevalence of SARS-CoV-2 based on different definitions of a case to ascertain the extent to which cases of SARS-CoV-2 may be underestimated by case-based surveillance.

**Methods:**

A survey on COVID-19 exposure, infection, and testing was administered to calculate point prevalence of SARS-CoV-2 among a diverse sample of cohort adults from February 8, 2022, to February 22, 2022. Three-point prevalence estimates were calculated among the cohort, as follows: (1) proportion positives based on polymerase chain reaction (PCR) and rapid antigen tests; (2) proportion positives based on testing exclusively with rapid at-home tests; and (3) proportion of probable undiagnosed cases. Test positivity and prevalence differences across booster status were also examined.

**Results:**

Among a cohort of 4328, there were a total of 644 (14.9%) cases. The point prevalence estimate based on PCR or rapid antigen tests was 5.5% (95% CI 4.8%-6.2%), 3.7% (95% CI 3.1%-4.2%) based on at-home rapid tests, and 5.7% (95% CI 5.0%-6.4%) based on the case definition of a probable case. The total point prevalence across all definitions was 14.9% (95% CI 13.8%-16.0%). The percent positivity among PCR or rapid tests was 50.2%. No statistically significant differences were observed in prevalence between participants with a COVID-19 booster compared to fully vaccinated and nonboosted participants except among exclusive at-home rapid testers.

**Conclusions:**

Our findings suggest a substantial number of cases were missed by case-based surveillance systems during the Omicron B.1.1.529 surge, when at-home testing was common. Point prevalence surveys may be a rapid tool to be used to understand SARS-CoV-2 prevalence and would be especially important during case surges to measure the scope and spread of active infections in the population.

## Introduction

Since the first US case of SARS-CoV-2 Omicron variant, B.1.1.529 (BA.1), was announced in December 2021 [1], its high transmissibility and immunogenetic characteristics led to dramatic increases in new cases and reinfections [2-4]. The rapid surge gave rise to community-wide spread across the country, straining testing capacities. In March 2022, the Centers for Disease Control and Prevention (CDC) updated their guidelines for monitoring community COVID-19 levels by tracking incident cases and hospital admissions and deaths to inform community prevention measures [5]; yet the number of new cases and the proportion positive among SARS-CoV-2 testers (percent positivity) are still used as local metrics to monitor SARS-CoV-2 transmission.

Both the number of reported cases and percent positivity are useful in monitoring changes in SARS-CoV-2 transmission; however, they inadequately capture the extent and spread of SARS-CoV-2 epidemic in the population due to the exclusion of undiagnosed and untested cases by standard surveillance [6-10]. To our knowledge, there is currently no mechanism in place in state and local jurisdictions in the United States for systematically capturing rapid at-home tests as part of a population-level indicator of SARS-CoV-2 spread. In Australia and the United Kingdom, for example, health departments put in place a reporting mechanism for individuals to report their rapid antigen test results. The extent to which the number of active SARS-CoV-2 infections is underestimated is likely to vary by geographic, sociodemographic, and economic factors associated with community and self-testing, in addition to temporal factors that drive test-seeking behaviors during a surge [11,12].

The objective of this analysis was to identify the extent to which cases of SARS-CoV-2 may be incomplete in standard case-based surveillance during the recent surge of the Omicron BA.1 variant. Using data from the national and longitudinal CHASING COVID cohort study, we compared point prevalence of SARS-CoV-2 infections captured by case-based surveillance based on polymerase chain reaction (PCR) and rapid antigen testing to a point prevalence estimated exclusively using rapid at-home SARS-CoV-2 tests as well as probable COVID-19 cases among nontesters. We also examined whether point prevalence differed by SARS-CoV-2 vaccine booster status.

## Methods

### Recruitment

The CHASING COVID Cohort study is a geographically and sociodemographically diverse sample of adults (18 and older), residing in the United States or its territories and enrolled into a prospective follow-up [13]. Study participants were originally recruited during the emergence of the US COVID-19 pandemic (March-April 2020) via social media (eg, Facebook) or via referral. Details of cohort recruitment and follow-up have been described elsewhere [13], but briefly, cohort participants have been prospectively followed with surveys occurring approximately every 3 months to capture a variety of measures, including COVID-19 symptoms, testing, hospitalizations, and adoption of nonpharmaceutical interventions. Survey materials and the timing of each survey are accessible on our website.

### Ethical Considerations

Informed consent was obtained at study enrollment. Participants receive US $10-15 in compensation for every standard study interaction and are entered into drawings for US $100 with 10 winners awarded. For brief study engagements, participants were entered into drawings with ten US $100 gift cards awarded. Study data are deidentified before analysis, and identifiable information remains on a secure server with limited access. The study protocol was approved by the Institutional Review Board at the City University of New York (protocol 2020-0256-PHHP).

### Point Prevalence Estimation

A questionnaire on recent COVID-19 exposure, infection, and testing was administered as the Omicron BA.1 surge was subsiding in the United States, in February 8-22, 2022. The questionnaire asked about the type and result of viral diagnostic tests taken in the past 7 days; the viral tests included PCR, rapid antigen, and rapid at-home tests. The survey collected information on experience in the previous 10 days with any COVID-19 symptoms for self, household, and close contacts, as well as exposure to a confirmed or probable COVID-19 case. COVID-19 symptoms were defined as having at least one of the following: fever of 100 degrees Fahrenheit or greater, cough, runny nose or nasal congestion, shortness of breath, sore throat, fatigue, muscle or body aches, headaches, loss of smell or taste, nausea, as well as vomiting or diarrhea [14].

We calculated 3 mutually exclusive prevalence estimates. First, prevalence was calculated as the proportion of participants reporting a positive result detected by PCR or rapid antigen tests and captured by case-based surveillance. Second, we calculated prevalence as the proportion of participants reporting a positive result using at-home rapid tests and who did not seek further testing, as well as prevalence of probable cases. A probable case, based on the Council of State and Territorial Epidemiologists case definition, did not receive any diagnostic test but reported SARS-CoV-2 symptoms and had an epidemiological linkage, either with a household member or close contact with infection [15]. We calculated the percent positivity as the proportion of positive cases among all testers.

Finally, we ascertained differences in point prevalence by booster status for the 3 case definitions. Booster status was measured as having received a SARS-CoV-2 booster between September 2, 2021, and January 11, 2022 [16]. Among participants who did not receive a booster dose, we further classified participants as fully, partially, or nonvaccinated with the SARS-CoV-2 vaccine.

### Statistical Analysis

Sociodemographic and health behaviors were described for testers and nontesters and by testing outcome. Pearson chi-squared test of independence was performed to assess group differences between testers and nontesters. To assess the effect of booster status on prevalence, we used a log-binomial model and presented adjusted prevalence ratios, adjusted for age, race or ethnicity, education, employment, smoking, essential worker status, and comorbidities. Analyses were performed using SAS, version 9.4 (SAS Institute).

## Results

A total of 4328 cohort participants (80% response rate among 5441 participants responding in 2021) completed the point prevalence questionnaire. Among the 841 testers, 396 (47.1%) had tested for SARS-CoV-2 on any diagnostic test (PCR, rapid antigen, or at-home rapid test; Table 1). Among the 3484 nontesters, 248 (7.1%) were probable cases. Testers were more likely to be >39 years old, gender nonbinary, college graduates, employed, and symptomatic, and to report close contact with a case, to have children in their households, to be in households with income above US $70,000, to have a prior SARS-CoV2 infection, to be at high risk for severe COVID-19 outcomes, and to have received a booster vaccine.

There was a total of 644 cases, among which 237 (36.8%) were positive based on point-of-care PCR or rapid antigen tests, 159 (24.7%) cases that were identified exclusively with at-home rapid tests, and 248 (38.5%) cases were probable cases. The prevalence estimate based on confirmed point-of-care PCR or rapid antigen tests was 5.5% (95% CI 4.8%-6.2%), of which 1.1% (95% CI 0.8%-1.4%) was based on rapid antigen tests only, 1.7% (95% CI 1.3%-2.2%) based on PCR tests only, and 2.6% (95% CI 2.2%-3.1%) based on both PCR and rapid antigen tests. The point prevalence based on those testing exclusively via rapid at-home tests was 3.7% (95% CI 3.1%-4.2%) and was 5.7% (95% CI 5.0%-6.4%) for probable cases. The total point prevalence was 14.9% (95% CI 13.8%-16.0%). The percent positivity among PCR or rapid antigen tests was 50.2%. Differences in SARS-CoV-2 prevalence among participants who had a COVID-19 booster versus those fully vaccinated and nonboosted participants were not statistically significant, except those diagnosed using at-home tests (adjusted prevalence ratio: 2.2, 95% CI 1.4%-3.4%; Table 2).

**Table 1:** Cohort characteristics by testing status and by test type (N=4328).

Characteristics			Total, n (%)		Nontesters, n (%)		Testers (any), n (%)		POC^a^ PCR^b^ test only, n (%)		POC rapid antigen test only, n (%)		With provider (POC) and at-home testers, n (%)		At-home rapid test only, n (%)		*P* value^c^	
Total			4328		3487 (80.6)		841 (19.4)		167 (3.9)		89 (2.1)		216 (5.0)		369 (8.5)		—^d^	
SARS-CoV-2 positive			644 (14.8)		—		—		—		—		—		—		—	
POC PCR or rapid antigen test cases			237 (5.5)		—		—		—		—		—		—		—	
Exclusive at-home test cases			159 (3.7)		—		—		—		—		—		—		—	
Probable cases			248 (5.7)		—		—		—		—		—		—		—	
**Age range**																		<.001
	18-29	826 (19.1)		636 (18.2)		190 (22.6)		56 (33.5)		15 (16.9)		51 (23.6)		68 (18.4)				
	30-39	1217 (28.1)		946 (27.1)		271 (32.2)		46 (27.5)		23 (25.8)		87 (40.3)		115 (31.2)				
	40-49	808 (18.7)		650 (18.6)		158 (18.8)		16 (9.6)		18 (20.2)		42 (19.4)		82 (22.2)				
	50-64	941 (21.7)		794 (22.8)		147 (17.5)		28 (16.8)		23 (25.8)		21 (9.7)		75 (20.3)				
	>65	536 (12.4)		461 (13.2)		75 (8.9)		21 (12.6)		10 (11.2)		15 (6.9)		29 (7.9)				
**Gender**																		.04
	Male	1913 (44.2)		1538 (44.1)		375 (44.6)		68 (40.7)		41 (46.1)		97 (44.9)		169 (45.8)				
	Female	2294 (53.0)		1862 (53.4)		432 (51.4)		90 (53.9)		46 (51.7)		112 (51.9)		184 (49.9)				
	Gender nonbinary	121 (2.8)		87 (2.5)		34 (4.0)		9 (5.4)		2 (2.3)		7 (3.2)		16 (4.3)				
**Race or ethnicity**																		.54
	Hispanic	657 (15.2)		527 (15.1)		130 (15.5)		28 (16.8)		19 (21.4)		40 (18.5)		43 (11.7)				
	Black non-Hispanic	385 (8.9)		308 (8.8)		77 (9.2)		11 (6.6)		21 (23.6)		21 (9.7)		24 (6.5)				
	Asian American or Pacific Islander	302 (7.0)		233 (6.7)		69 (8.2)		18 (10.8)		5 (5.6)		20 (9.3)		26 (7.1)				
	White non-Hispanic	2824 (65.5)		2287 (65.6)		537 (63.9)		102 (61.1)		43 (48.3)		129 (59.7)		263 (71.3)				
	Other	160 (3.4)		132 (3.8)		28 (3.3)		8 (4.8)		1 (1.1)		6 (2.8)		13 (3.5)				
**Income (US $)**																		.009
	<35,000	1115 (25.8)		937 (26.9)		178 (21.2)		40 (24.0)		23 (25.8)		48 (22.2)		67 (18.2)				
	35,000-49,000	479 (11.1)		389 (11.2)		90 (10.7)		19 (11.4)		8 (9.0)		30 (13.9)		33 (8.9)				
	50,000-69,000	638 (14.7)		513 (14.7)		125 (14.9)		26 (15.6)		14 (15.7)		38 (17.6)		47 (12.7)				
	70,000-99,000	737 (17.0)		592 (17.0)		145 (17.2)		28 (16.8)		22 (24.7)		25 (11.6)		70 (19.0)				
	>100,000	1236 (28.6)		961 (27.6)		275 (32.7)		45 (27.0)		21 (23.6)		67 (31.0)		142 (38.5)				
	Missing or unknown	123 (2.8)		95 (2.7)		28 (3.3)		9 (5.4)		1 (1.1)		8 (3.7)		10 (2.7)				
**Education**																		.03
	<High school	59 (1.4)		51 (1.5)		8 (1.0)		3 (1.8)		1 (1.1)		2 (0.9)		2 (0.5)				
	High school	383 (8.9)		324 (9.3)		59 (7.0)		12 (7.2)		6 (6.7)		20 (9.3)		21 (5.7)				
	Some college	1089 (25.2)		892 (25.6)		197 (23.4)		38 (22.8)		31 (34.8)		52 (24.1)		76 (20.6)				
	College graduate	2797 (64.6)		2220 (63.7)		577 (68.6)		114 (68.3)		51 (57.3)		142 (65.7)		270 (73.2)				
**Employment**																		.001
	Employed	1704 (39.4)		1343 (38.5)		361 (42.9)		65 (38.9)		35 (39.3)		91 (42.1)		170 (46.1)				
	Out of work	615 (14.2)		522 (15.0)		93 (11.1)		13 (7.8)		11 (12.4)		22 (10.2)		47 (12.7)				
	Student	250 (5.8)		187 (5.4)		63 (7.5)		25 (15.0)		4 (4.5)		19 (8.8)		15 (4.1)				
	Other or unknown	1759 (40.6)		1435 (41.2)		324 (38.5)		64 (38.3)		39 (43.8)		84 (38.9)		137 (37.1)				
**Children in household**																		.57
	Yes	1163 (26.9)		915 (26.2)		248 (29.5)		32 (19.2)		29 (32.6)		85 (39.4)		102 (27.6)				
	No	3165 (73.1)		2572 (73.8)		593 (70.5)		135 (80.8)		60 (67.4)		131 (60.7)		267 (72.4)				
**Vaccination status**																		<.001
	Boosted	2810 (64.9)		2191 (62.8)		619 (73.6)		121 (72.5)		51 (57.3)		154 (71.3)		293 (79.4)				
	Fully vaccinated	1029 (23.8)		875 (25.1)		154 (18.3)		35 (21.0)		25 (28.1)		40 (18.5)		54 (14.6)				
	Partially vaccinated	81 (1.9)		63 (1.8)		18 (2.1)		1 (0.6)		4 (4.5)		6 (2.8)		7 (1.9)				
	Not vaccinated	408 (9.4)		358 (10.3)		50 (6.0)		10 (6.0)		9 (10.1)		16 (7.4)		15 (4.1)				
**Prior COVID-19 infection**																		.01
	Yes	696 (16.1)		545 (15.6)		151 (18.0)		26 (15.6)		23 (25.8)		56 (25.9)		46 (12.5)				
	No	3632 (83.9)		2942 (84.4)		690 (82.1)		141 (84.4)		66 (74.2)		160 (47.2)		323 (87.5)				
**COVID-19–like symptoms**																		<.001
	Yes	760 (17.6)		434 (12.5)		326 (38.8)		44 (26.4)		25 (28.1)		116 (53.7)		141 (38.2)				
	No	3568 (82.4)		3053 (87.6)		515 (61.2)		123 (73.7)		64 (71.9)		100 (46.3)		228 (61.8)				
**High risk status^e^**																		.003
	Yes	2191 (50.6)		1804 (51.7)		387 (46.0)		74 (44.3)		53 (59.3)		101 (46.8)		159 (43.1)				
	No	2137 (49.4)		1683 (48.3)		454 (54.0)		93 (55.7)		36 (40.5)		115 (53.2)		210 (56.9)				
**Close contact with confirmed case**																		<.001
	Yes	630 (14.6)		336 (9.6)		294 (35.0)		51 (30.5)		28 (31.5)		102 (47.2)		113 (30.6)				
	No	3698 (85.4)		3151 (90.4)		547 (65.0)		116 (69.5)		61 (68.5)		114 (52.8)		256 (69.4)				

^a^POC: point-of-care.

^b^PCR: polymerase chain reaction.

^c^*P* value corresponds to cohort group differences between testers and nontesters.

^d^Not applicable.

^e^Essential worker, >60 years old, smoker, and reported comorbidities.

**Table 2:** Point prevalence estimates by vaccination status, February 2-22, 2022 (N=4328).

Variable	Point prevalence											
	Cases identified with PCR^a^ or rapid antigen tests			Cases identified with at-home rapid tests			Probable cases			Total prevalence		
	N	% (95% CI)	N		% (95% CI)	N		% (95% CI)	N		% (95% CI)	
Total	237	5.5 (4.8-6.2)	159		3.7 (3.1-4.3)	248		5.7 (5.0-6.4)	644		14.9 (13.8-15.9)	
Boosted	150	5.3 (4.5-6.2)	120		4.3 (3.5-5.0)	132		4.7 (3.9-5.4)	402		14.3 (13.0-15.6)	
Nonboosted or fully vaccinated	59	5.7 (4.3-7.2)	22		2.1 (1.3-3.0)	67		6.5 (5.0-8.0)	148		14.3 (12.2-16.5)	
Nonboosted or partially vaccinated	7	8.6 (2.4-14.9)	4		4.9 (0.1-9.8)	6		7.4 (1.6-13.2)	17		21.0 (11.9-30.0)	
No vaccine or unknown	21	5.1 (3.0-7.3)	13		3.2 (1.5-4.9)	43		10.5 (7.5-13.5)	77		18.9 (15.1-22.7)	
Boosted vs fully vaccinated^b^	237	1.1 (0.84-1.56)^c^	159		2.2 (1.4-3.4)^c^	248		0.8 (0.6-1.1)^c^	644		1.1 (1.0-1.4)^c^	

^a^PCR: PCR: polymerase chain reaction.

^b^Model adjusted for race or ethnicity, age, education, employment, smoking, essential worker status, and comorbidities.

^c^Adjusted prevalence ratio.

## Discussion

### Principal Findings

Our findings showed a high prevalence of SARS-CoV-2 in our cohort during the decline of the Omicron BA.1 wave in the United States in February 2022. Our results are not directly comparable to US national estimates as CDC’s COVID-19 tracker only captures test positive results based on PCR tests and does not include point-of-care antigen tests as done at some local or state levels [9]. Our study suggests a substantial proportion of cases would be missed by standard case-based surveillance systems during the Omicron BA.1 wave, when at-home testing was common [17]. The number of cases detected by case-based surveillance was lower than the total number of cases in our cohort, while the percent positivity was higher than the total prevalence based on all definitions. The underestimated case burden and overestimated percent positivity illustrates the limitations of case-based surveillance, and the extent to which current metrics used to monitor SARS-CoV-2 infection may be incomplete. In addition, we found the characteristics among testers differed considerably from nontesters, underscoring the limitations around case-based surveillance data for understanding the epidemiology and any disparities around SARS-CoV-2 burden and community transmission.

The CDC issued recommendations that shifted away from positivity rates and toward the use of hospital admission and death rate. While hospital admission and death rates better capture disease severity, they lag community transmission by weeks and are of limited use in providing early warning for active community infection. By contrast, and while state and local health departments continue to use metrics such as incident cases and test positivity, population-based surveys may be deployed frequently to capture spread and susceptibility to inform more effective mitigation measures.

We found no statistically significant differences in SARS-CoV-2 prevalence by booster status among those who tested exclusively using at-home rapid tests. These findings may be driven by higher testing frequency as was observed among boosted adults compared to those nonboosted but fully vaccinated adults. In general, our findings align with evidence from studies that show that standard SARS-CoV-2 vaccines plus the additional booster dose offer limited additional protection against symptomatic and asymptomatic infection from the Omicron BA.1 variant; however, boosters have been shown to be effective at reducing severe outcomes such as COVID-19 hospitalizations and deaths, which we did not assess [18,19].

### Limitations

Our method had key limitations. First, we measured infection and testing outcomes with self-report, which is prone to misclassification bias. In lieu of biomarker data, we classified an undiagnosed and untested case based on any self-reported COVID-19 symptoms and on contact with a confirmed or probable case, which might lead to an overestimation of true infection status. Furthermore, the latest booster status information on participants was collected before January 11, 2022, potentially missing booster information on those who received a booster between January 11 and the survey date. Additionally, our results for booster dose effectiveness did not adjust for the timing of the booster or consider previous infection history.

Our survey questionnaire consisted of fewer than 20 questions and required less than 10 minutes to complete. Our survey was not intended to be representative of the US population as it aimed to capture the extent of which surveillance data are incomplete and representative, and probability-based point prevalence surveys may be used in tandem with surveillance metrics to rapidly understand local spread and to measure the scope of active infections in the population [20-22] and other highly pertinent epidemiological information. At this stage of the pandemic, the application of low-cost and low-resource intensive tools such as routine population-based surveys may have a large impact on effectively informing the control and prevention of community spread of SARS-CoV-2.

## Abbreviations

BA.1: B.1.1.529
CDC: Centers for Disease Control and Prevention
PCR: polymerase chain reaction
